# Splice variation in the cytoplasmic domains of myelin oligodendrocyte glycoprotein affects its cellular localisation and transport^1^

**DOI:** 10.1111/j.1471-4159.2007.04687.x

**Published:** 2007-09

**Authors:** Louise H Boyle, James A Traherne, Gemma Plotnek, Rosemary Ward, John Trowsdale

**Affiliations:** Department of Pathology, Cambridge Institute of Medical Research, University of Cambridge Addenbrooke’s Hospital, Cambridge, UK

**Keywords:** alternative splicing, cellular trafficking, human, multiple sclerosis, myelin

## Abstract

Although myelin oligodendrocyte glycoprotein is a candidate autoantigen in multiple sclerosis, its function remains unknown. In humans, mRNA expressed by the myelin oligodendrocyte glycoprotein gene is alternatively spliced resulting in at least nine unique protein isoforms. In this study, we investigated the sub-cellular localisation and membrane trafficking of six isoforms by cloning them into mammalian expression vectors. Confocal microscopy revealed that these protein products are expressed in different cellular compartments. While two full-length isoforms (25.6 and 25.1) are expressed at the cell surface, three alternatively spliced forms (22.7, 21.0 and 20.5) have a more intracellular distribution, localising to the endoplasmic reticulum and/or endosomes. Isoform 16.3, which lacks a transmembrane domain, is secreted. A switch in the sub-cellular localisation of myelin oligodendrocyte glycoprotein may have profound effects on receptor:ligand interactions and consequently the function of the protein. The structural features of the alternative isoforms and their differential, sub-cellular expression patterns could dictate the exposure of major immunogenic determinants within the central nervous system. Our findings highlight myelin oligodendrocyte glycoprotein splicing as a factor that could be critical to the phenotypic expression of multiple sclerosis.

Multiple sclerosis (MS) is a common, chronic disorder of the central nervous system (CNS) often triggered in young adults. Pathogenesis is characterised by inflammation, destruction of the myelin sheath and axonal degeneration, resulting in a disabling condition. MS is thought to be an immune mediated disease (for review, see Sospedra and Martin 2005). T-cell responses to many myelin proteins including myelin basic protein, proteolipid protein, and myelin oligodendrocyte glycoprotein (MOG) are observed in MS patients and are thought to play a role in the pathogenesis of the disease. Despite constituting only ∼0.05% of the total myelin protein (Johns and Bernard 1999), MOG is a highly significant B cell autoantigen in MS, with antibodies against MOG contributing to myelin destruction (Schluesener *et al.* 1987; Linington *et al.* 1988; Litzenburger *et al.* 1998; Storch *et al.* 1998). Therefore, MOG is a unique antigen as it triggers both inflammatory T-cell responses and demyelinating B-cell responses (Iglesias *et al.* 2001).

Despite the strong evidence of MOG as an autoantigen, its precise function remains unknown (Johns and Bernard 1999). MOG is known to dimerise, however, its interactions with other cellular proteins are undefined. MOG is generally thought of as a membrane-spanning glycoprotein found on the outermost surface of the myelin sheath (Brunner *et al.* 1989; Scolding *et al.* 1989). It has an unusual protein topology; after the extracellular IgV domain, MOG contains a hydrophobic transmembrane (TM) domain, a cytoplasmic loop, a second hydrophobic membrane associate region, followed by a cytoplasmic tail (Kroepfl *et al.* 1996; della Gaspera *et al.* 1998) (Fig. 1).

**Fig. 1:**
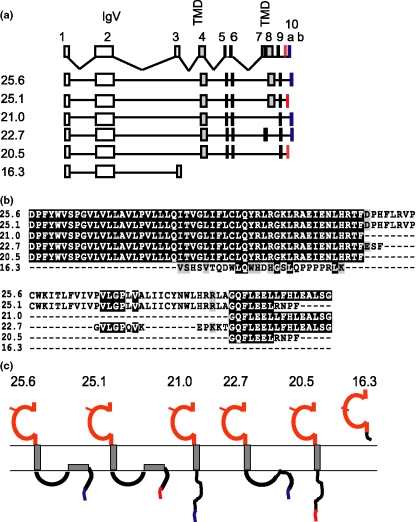
Composition of investigated myelin oligodendrocyte glycoprotein (MOG) transcripts and their products. (a) Exon structure of the six MOG transcripts of predicted mRNA NM_002433 (25.6), NM_206809 (25.1), NM_206810 (21.0), NM_206811 (22.7), NM_206812 (20.5) and NM_206814 (16.3). (b) Alignment of amino acids encoded by exons 3–10 of MOG transcripts. The sequences were aligned using Clustal W software. (c) Schematic representations of the MOG isoforms (as suggested by Hilton *et al.* 1995; Pham-Dinh *et al.* 1995; Ballenthin and Gardinier 1996). Protein molecular weights are shown in kDa above each form.

Myelin oligodendrocyte glycoprotein is encoded on chromosome 6 in the major histocompatibility complex (Pham-Dinh *et al.* 1993), a region of the genome encoding many molecules important in the processing and presentation of antigens to the immune system (Trowsdale 2001). The mRNA product of the human MOG gene, but not the mouse or rat analogues (Gardinier *et al.* 1992; Daubas *et al.* 1994), can undergo significant differential splicing, potentially resulting in 17 protein isoforms (Hilton *et al.* 1995; Pham-Dinh *et al.* 1995; Ballenthin and Gardinier 1996; Delarasse *et al.* 2006). The MOG splice variants are capable of being translated into functional proteins, hence, it is important to consider the roles that individual isoforms play in the CNS, and in MS. The expression of alternative MOG isoforms could modulate protein interactions, the activity of the major isoform, the sub-cellular localisation of MOG, and consequently myelin maintenance.

The major splice forms investigated to date are shown in Table 1. Alpha 1, the major variant investigated thus far, and beta 1 are full-length isoforms, encoding proteins of 25.1 and 25.6 kDa, respectively. These two isoforms vary only in their C-terminal amino acids encoded by exon 10; the C-terminal amino acids encoded by the 10A exon are RNPF, while those encoded by the 10B exon are LFHLEALSG (Fig. 1). Splice forms alpha 4 and beta 4 utilise exon 3, an *Alu*-encoded sequence that contains a stop codon. As none of the later exons contribute to the protein produced, these two variants result in the same truncated protein product, MOG 16.3 that lacks a TM domain. This product is predicted to be secreted. The other variants lack exon 8, which is believed to encode a membrane associated region, and use alternative intron/exons boundaries to produce a variety of alternative cytoplasmic tails (Fig. 1). The resulting protein isoforms are predicted to be 22.7, 22.2, 21.0, 20.5 and 20.2 kDa in size (see Table 1).

**Table 1:** MOG splice isoforms

	Exon composition										
		
Variant	1	2	3	4	5	6	7	8	9	10	Isoform name/ protein size
Alpha 1	√	√		√	√	√		√	√	A	MOG 25.1 +
Alpha 2	√	√		√	√	√			√	A	MOG 20.5 +
Alpha 3	√	√		√	√	√	√		√	A	MOG 22.2
Alpha 4	√	√	√*	√	√	√		√	√	A	MOG 16.3 +
Beta 1	√	√		√	√	√		√	√	B	MOG 25.6 +
Beta 2	√	√		√	√	√				B	MOG 20.2
Beta 3	√	√		√	√	√	√		√	B	MOG 22.7 +
Beta 4	√	√	√*	√	√	√		√	√	B	MOG 16.3 +
Beta 5	√	√		√	√	√			√	B	MOG 21.0 +

* Exon 3 encodes a stop codon therefore the protein product is truncated and none of its later exons contribute to the amino acid sequence.

+ Isoforms selected for further investigation in this study.

As the cytoplasmic tails of many transmembrane proteins often contain motifs important in cellular transport, we hypothesised that MOG variants with alternative cytoplasmic tails may traffic differently. In this study, we investigated the trafficking of six of these isoforms; MOG 25.6, 25.1, 22.7, 21.0, 20.5 and 16.3 (Fig. 1). We report that variation in the cytoplasmic tails of MOG isoforms has profound affects on its cellular localisation and transport.

## Materials and methods

### Plasmids

The alpha 1 variant (MOG 25.1) was isolated from human brain cDNA (Stratagene) by PCR amplification using primers 5′ CCTGAGATCTGGAACAGTAGAGATGGCAAGC 3′ with a *Bgl*II site and 5′ GGACACCGTTGAAGGGATTTCGTAGC 3′ containing an *Age*I site. This was subsequently cloned into pcDNA3.0 (Invitrogen Life Technologies, Paisley, UK) containing a cytomegalovirus promoter. We constructed MOG variants beta 1 (MOG 25.6), beta 3 (MOG 22.7), beta 5 (MOG 21.0), alpha 2 (MOG 20.5) and alpha 4/beta 4 (MOG 16.3) from the alpha 1 variant plasmid by sequential PCR amplification using 5′ tailed antisense primers. The PCR primer sequences were as follows: MOG25.6-R 5′CAGGACCGGTGCCAGAGAGGGCTTCCAGGTGGAAGAGTAGCTCTTCAAGGAATTGCCCTGCTAGTC3′, MOG21.0-R 5′CCACACCGGTGCCAGAGAGGGCTTCCAGGTGGAAGAGTAGCTCTTCAAGGAATTGCCC3′, MOG22.7-R1 5′CCACACCGGTCTTTTTTGGTTCCTTAACCTGGGGTCCTAGAACACCAAAGGACTCAAAAGTCCGGTGGAGATTCTC3′, MOG22.7-R2 5′CCACACCGGTGCCAGAGAGGGCTTCCAGGTGGAAGAGTAGCTCTTCAAGGAATTGCCCTGTCTTTTTTGGTTCCTTAAC3′, MOG20.5-R 5′GGACACCGGTGAAGGGATTTCGTAGCTCTTCAAGGAATTGCCCAAAAGTCCGGTGGAGATTCTC3′, MOG16.3-R1 5′CCACACCGGTGTGCCACTGCAACCAATCCTGGGTCACAGAGTGAGACACTTCTACTTTCAATTCCATTGCTG3′, MOG16.3-R2 5′CCACACCGGTCTTGAGCCTGGGAGGTGGAGGCTGCAATGAGCCATGATCGTGCCACTGCAACCAATCCTGG3′. All variants were cloned into pcDNA3.0 and were also cloned into a mammalian cell vector (provided by Alison Gillingham, MRC-LMB, Cambridge, UK) containing a C-terminal green fluorescent protein (GFP) tag. Sequence analysis using BigDye technology was preformed to ensure all vectors were correct and in-frame.

### Immunofluorescence

The human oligodendrocyte cell line MO3.13 (McLaurin *et al.* 1995) (a kind gift from Neil Cashman, University of British Columbia, Canada) and HeLa cells were grown on glass coverslips in Dulbecco’s modified Eagle’s medium (Invitrogen Life Technologies) supplemented with 10% heat-inactivated foetal calf serum, 20 mmol/L HEPES, 2 mmol/L glutamine, 100 μg/mL streptomycin, and 100 U/mL penicillin. MO3.13 and HeLa cells were transiently transfected using FuGene 6 transfection reagent (Roche, Lewes, UK) or HeLa Monster (Mirus, Madison, WI, USA), respectively. Approximately, 36 h post-transfection cells were stained for immunofluorescence. MO3.13 cells were fixed and permeabilised in acetone. HeLa cells were fixed in 4% paraformaldehyde and permeabilised with 0.5% (v/v) Triton X-100 in phosphate-buffered saline (PBS). After blocking in 20% (v/v) heat-inactivated foetal calf serum, 0.5% (v/v) Tween 20 in PBS, cells were stained with primary antibodies. To detect untagged MOG, cells were stained with the MOG specific mAb 8.18C5 (a kind gift from Robert Harris, Karolinska Institutet, Stockholm, Sweden). In addition, antibodies specific for cellular compartments including the plasma membrane (Rat anti-CD44, PharMingen, Oxford, UK), early endosomes (mouse anti-EEA1, BD Transduction Laboratories, Oxford, UK), endosomes (anti-Vps26, a kind gift from Matthew Seaman, University of Cambridge, UK), the endoplasmic reticulum (ER) (rabbit anti-calreticulin, Calbiochem, San Diego, CA, USA), lysosomes (mouse anti-LAMP2, Developmental Studies hybridoma bank, University of Iowa, IA, USA), and the *cis*-Golgi (mouse anti-GM130, BD Transduction Laboratories) were also used. Staining with primary antibodies was subsequently detected with species-specific Alexa secondary antibodies (Molecular Probes, Paisley, UK). Cells were mounted in fluoromount-G (Southern Biotechnology Associates, Birmingham, AL, USA) and images obtained using a Zeiss confocal microscope (Carl Zeiss Microimaging GmbH, Hamburg, Germany).

To track MOG internalised into cells, antibody internalisation experiments were performed. 36 h post-transfection cells were continuously incubated with mAb 8.18C5 for 2 h at 37°C to allow staining of MOG at the cell surface and detection of any MOG internalised. The cells were washed of excess antibody, fixed and permeabilised as above, then stained with antibodies specific to the endocytic/ lysosomal pathway. After washing, primary antibodies were detected using species-specific Alexa secondary antibodies.

### Radiolabelling

Around 36 h post-transfection, cells were harvested, washed in PBS then starved for 1 h at 37°C in methionine/cysteine-free RPMI 1640 (Sigma, Poole, UK) supplemented with 2 mmol/L glutamine, 5% dialysed foetal calf serum, and 10 mmol/L HEPES. Cells were labelled with 1 mCi [^35^S] methionine and [^35^S] cysteine Pro-mix (Amersham Pharmacia; GE Healthcare, Little Chalfont, UK)/10^7^ cells for 10 min at 37°C. Cells were chased in culture medium supplemented with excess unlabelled cysteine/methonine for 0–240 min, harvested, then washed in ice-cold PBS.

### Immunoprecipitation

Radiolabelled cells were lysed in 1% Triton X-100 lysis buffer [150 mmol/L NaCl, 20 mmol/L Tris, 1 mmol/L EDTA, 5 mmol/L MgCl_2_ with 1 mmol/L phenylmethylsulfonyl fluoride and protease inhibitors (Roche)]. After centrifugation at 17 000 *g* for 10 min to remove cell nuclei and debris, supernatants were pre-cleared on a mixture of sepharose and protein A sepharose beads (Amersham Biosciences) for 1 h at 4°C. To immunopreciptiate MOG isoforms, 2 × 10^6^ cells were incubated with 2 μg 8.18C5 for 1 h on ice, before incubation with protein A sepharose (Amersham Biosciences) for 1 h at 4°C. Unbound proteins were removed by washing the beads three times in 0.5% Triton X-100 lysis buffer, before bound proteins were eluted in sodium dodecyl sulphate sample buffer with 100 mmol/L beta-mercaptoethanol. Proteins were then digested with 1000 U of Endo Hf (New England Biolabs, Beverly, MA, USA) for 2 h in G5 buffer at 37°C to allow quantification of the amount of MOG exported through the secretory pathway to the medial Golgi.

### Gel electrophoresis and immunoblotting

To detect any secreted MOG, GFP-transfected cell culture supernatants were collected, any cells were removed by centrifugation, then the clarified medium was mixed with sample buffer. Whole cell lysates were made in sample buffer. Protein samples were separated by sodium dodecyl sulphate-polyacrylamide gel electrophoresis. For metabolically labelled proteins, gels were fixed in 40% methanol, 12% acetic acid, and dried before images were obtained using a phosphor screen (Perkin-Elmer, Waltham, MA, USA) and storm scanner (Molecular Dynamics; Amersham Pharmacia) or on film. For immunoblotting, proteins were transferred to an Immobolin membrane (Millipore) and blocked in 5% (w/v) dried milk, 0.05% (v/v) Tween 20 in PBS. Membranes were incubated with a rabbit polyclonal to GFP (Abcam, Cambridge, UK) followed by species-specific horseradish peroxidase secondary Abs (DAKO, Ely, UK), before detection by enhanced chemiluminescence reagent (Amersham Biosciences).

### Screening for MOG RNA expression

Brain samples from MS patients and controls were obtained by the UK Multiple Sclerosis Tissue Bank, Imperial College London by written consent and approved by Multicentre Research Ethics Committee (MREC/02/2/39). The institute’s general safety committee and Peterborough and Fenland Local Research Ethics Committee approved experimental procedures. RNA was extracted using TRI REAGENT method (Sigma). In addition, brain RNA from control samples from Stragene and Clonetech were included in this study. RNA samples were DNase I treated prior to reverse transcription using a DNA-free kit (Ambion, Huntingdon, UK). First strand cDNA was synthesised from 4 μg of extracted RNA using Transcriptor Reverse Transcriptase (Roche). The unique combinations of exon–exon pairings within the splice variant cDNA sequences were used to design specific PCR assays for the analysis of their expression. The PCR primer sequences were as follows: EX2–3 F 5′GGAATTGAAAGTAGAAGTGTCTCAC3′, EX2–4 F 5′GCAATGGAATTGAAAGTAGAAGAT3′, EX6–9 F 5′CGGACTTTTGGGCAATTC3′, EX6–10BR 5′TTCCAGGTGGAAGACAAAAG3′, EX7–9R 5′CTTCAAGGAATTGCCCTGTC3′, EX8–9R 5′CTTCAAGGAATTGCCCTGCT3′, EX9–10BR 5′GGCTTCCAGGTGGAAGAGTA3′, EX10A-10BR 5′TTCCAGGTGGAAGACTGGAG3′. The primer pair combinations for each splice variant-specific PCR (svPCR) assay were: MOG 25.1/MOG 25.6 (full-length variants; alpha/beta 1) EX2–4 F with EX8–9R, MOG 20.5 (alpha 2) EX6–9 F with EX10A-10BR, MOG 20.2 (beta 2) EX2–4 F with EX6–10BR, MOG 22.2/MOG 22.7 (alpha/beta 3) EX2–4 F with EX7–9R, MOG 16.3 (alpha 4) EX2–3 F with EX10A-10BR, MOG 16.3 (beta 4) EX2–3 F with EX9–10BR. An assay was not achieved for MOG 21.0 (beta 5). Amplification was performed in a volume of 20 μL containing 200 μmol/L dNTP, 500 nmol/L primer, 2.5 mmol/L MgCl_2_, 10 mmol/L Tris–HCl (pH 8.3), 50 mmol/L KCl, 0.3U AmpliTaq Gold polymerase (Applied Biosystems, Foster City, CA, USA), and 1 μL of the cDNA reaction. Cycling was performed as follows: 12 min at 95°C; cycles of 95°C for 25 s, 58°C or 62°C for 45 s, 72°C for 30 s, and a final extension step of 30 s at 72°C. PCR products were electrophoresed in 1% or 3% agarose gels containing ethidium bromide, and predicted size products were visualized under UV light.

Expected products sizes for Alpha/Beta 1, Alpha 2, Alpha/Beta 3, Alpha 4, Beta 2, Beta 4 were 310, 305, 245, 718, 196, 487 bp, respectively. The PCR products were sequenced to confirm their identity. Plasmid controls were also used to verify PCR specificities. A PCR for glyceraldehyde-3-phosphate dehydrogenase was used as a control for cDNA synthesis.

## Results

### Isoforms of MOG exhibit different rates of export from the endoplasmic reticulum

**Fig. 2:**
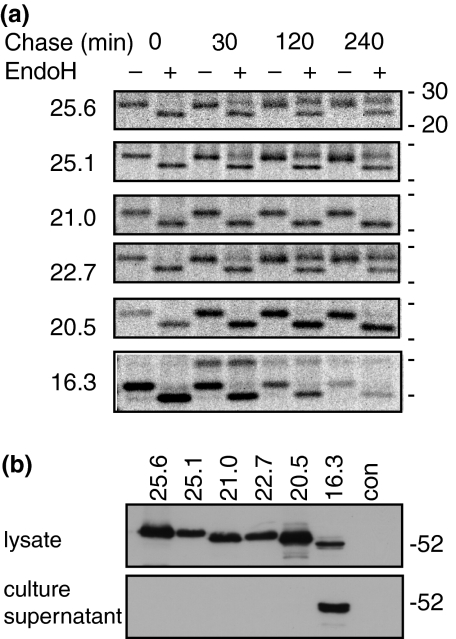
Rates of export of myelin oligodendrocyte glycoprotein (MOG) isoforms. (a) HeLa cells transiently transfected with the various MOG isoforms were pulse labelled for 10 min and chased for 0–240 min, followed by immunoprecipitation of MOG with 8.18C5, digestion with EndoH and electrophoresis on 12% acrylamide gels. Size markers are shown on right-hand side (top line, 30 kDa; bottom, 20 kDa). (b) Western blots of cellular lysates and culture supernatants of HeLa cells transiently expressing the MOG proteins tagged at the C-terminus with GFP. MOG proteins were detected by blotting with rabbit anti-GFP. 52 kDa marker is shown.

To determine if variation in the cytoplasmic tails of MOG isoforms affects trafficking, we initially investigated the rate of export of MOG. To this end, the different MOG isoforms were cloned into expression vectors and transiently expressed in HeLa cells. After metabolic labelling of MOG transfected HeLa cells, samples were chased for 0–240 min, and then the MOG isoforms were immunoprecipitated with the MOG-specific mAb 8.18C5. The samples were subsequently treated with Endo H, an enzyme that does not cleave processed N-linked sugars, a modification indicative of transit to the medial Golgi. This revealed that export of isoform 25.6, 25.1 and 22.7 to the Golgi apparatus was similar, with each protein gaining ∼50% Endo H resistance in 4 h (Fig. 2a). In contrast, isoform 21.0 remained totally Endo H sensitive (Fig. 2a), suggesting that the majority of MOG 21.0 protein was retained in the ER. Similar results were obtained with MOG 20.5, with the majority of the protein remaining Endo H sensitive after 4 h (Fig. 2a). The three exported forms, 25.6, 25.1 and 22.7 contain a potential membrane-associated region in their cytoplasmic tails, specifically sharing a motif VLGP×V. In contrast, the two isoforms that exhibited slower export, 21.0 and 20.5, lack this region/motif. These results suggest that the cytoplasmic tail encoding the VLGP×V motif is essential for the export of MOG isoforms from the ER.

### MOG isoform 16.3 is secreted

MOG 16.3 is predicted to be secreted as it lacks the TM domain. The pulse/chase analysis suggested that MOG 16.3 was indeed secreted from transfected HeLa cells since the protein disappeared over the chase time to very low levels after 4 h (Fig. 2a). The secretion of MOG 16.3 was confirmed by the detection of GFP tagged MOG 16.3, but not the other isoforms, from culture supernatant of transfected HeLa cells by western blotting (Fig. 2b). Interestingly, a novel band of ∼30 kDa was associated with the MOG 16.3 isoform at the 30 min chase period. This band may represent a protein ligand that uniquely associates with MOG 16.3 (Fig. 2a). We are currently attempting to identify this molecule.

### MOG 25.6 and 25.1 are expressed on the cell surface

**Fig. 3:**
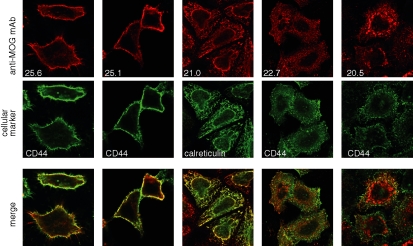
Myelin oligodendrocyte glycoprotein (MOG) isoforms are expressed at different subcellular sites in HeLa cells. Confocal micrographs of HeLa cells transiently expressing MOG 25.6, 25.1, 21.0, 22.7 and 20.5. Cells were fixed, permeabilised and stained with the MOG-specific antibody 8.18C5 and markers for the cell surface (CD44) or the ER (calreticulin). Staining was detected with Alexa secondary antibodies.

Next, we investigated the specific cellular distribution of MOG isoforms containing the TM domain via confocal microscopy using the MOG-specific mAb 8.18C5. As expected, the major isoform of MOG previously investigated, MOG 25.1 was found on the cell surface when transiently expressed in HeLa cells, where it co-localised with the cell surface marker CD44 (Fig. 3). Similarly, MOG 25.6 was also expressed at the cell surface (Fig. 3).

### MOG 21.0 is predominately expressed in the endoplasmic reticulum

In contrast to 25.6 and 25.1, the shorter isoforms 21.0, 22.7 and 20.5 clearly demonstrated a more intracellular pattern of expression (Fig. 3). MOG 21.0 had a reticular pattern of expression, which co-localised perfectly with the ER marker calreticulin (Fig. 3). Therefore, MOG 21.0 was mainly retained in the ER with very little cell surface expression. This result fits with the slow rates of export of MOG 21.0 as observed in Fig. 2a.

### MOG 22.7 is internalised into the endocytic system

**Fig. 4:**
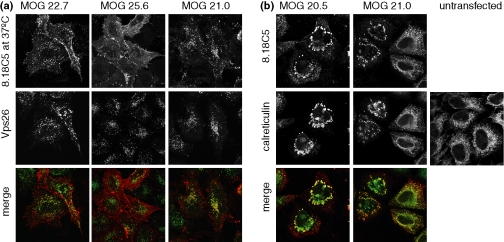
Endoplasmic reticulum (ER) and endosomal localisation of specific isoforms. (a) MOG 22.7 and MOG 21.0 are internalised into endosomes. Confocal micrographs of HeLa cells expressing MOG 22.7, 25.6 or 21.0 incubated with 8.18C5 for 2 h at 37°C to allow internalisation of MOG. Cells were then fixed, permeabilised, and stained for the endocytic marker Vps26. Staining was detected with Alexa secondary antibodies. (b) MOG 20.5 and MOG 21.0 are retained in the ER. Confocal micrographs of HeLa cells transiently expressing MOG 20.5 and 21.0. Cells were fixed, permeabilised and stained with the MOG-specific antibody 8.18C5 and the ER marker calreticulin.

MOG 22.7 was capable of being expressed at the cell surface showing co-localisation with CD44 (Fig. 3). However, much of the protein was found in distinct vesicles. To characterise the type of vesicle more thoroughly we compared the localisation of MOG 22.7 with a variety of cell markers including those for the ER (calreticulin), *cis*-Golgi (GM130), *trans*-Golgi (TGN-46), endosomes (Vps26), mitochondria (ATP synthase), early endosomes (EEA1) and lysosomes (LAMP2). MOG 22.7 co-localised with markers of the endocytic pathway including EEA1, Vps26 and LAMP2 (data not shown). As MOG 22.7 showed a similar rate of export to the Golgi as the two surface expressed isoforms MOG 25.6 and 25.1, and was expressed at the cell surface, we suspected that MOG 22.7 had been internalised from the cell surface into the endocytic vesicles. To visualise this we incubated MOG transfected cells with 8.18C5 at 37°C for 2 h to detect only surface expressed MOG and to follow its internalisation over the time period. The cells were then fixed and permeabilised, and stained with endocytic markers. Using this method, we clearly detected MOG 22.7 at the cell surface and observed its internalisation into the endocytic system, where it co-localised significantly with the endocytic marker Vps26 (Fig. 4a). As a control, we examined the internalisation of the surface expressed MOG 25.6, finding very little of this MOG isoform co-localising with Vps26 (Fig. 4a). Therefore, after export through the Golgi to the cell surface, MOG 22.7 is internalised into the endocytic system.

To a lesser extent, MOG 21.0 could also be detected in the endocytic system using this experimental procedure (Fig. 4a). Thus, although this isoform is mainly expressed in the ER, any MOG 21.0 released to the cell surface was internalised into endosomes. As the two isoforms internalised into endosomes both have the 10B tail these results suggest that an endosomal-targeting motif is present in the 10B tail. However, such a motif is only functional in the absence of exon 8, as isoform 25.6 was not significantly internalised into endosomes.

### MOG 20.5 is predominately expressed in the endoplasmic reticulum

The MOG 20.5 was found at low levels at the cell surface where it co-localised with CD44 (Fig. 3). However, this isoform was mainly found in large “donut-shaped” structures, which often accumulated around the nucleus (Fig. 3). Initially, we thought that these were vesicles of the endocytic/lysosomal system. However, staining with markers for these compartments revealed only little co-localisation. To our surprise, we found that these “donut-shaped” structures were actually the ER, as shown with co-localisation with the ER marker calreticulin (Fig. 4b). These results fit with the slow rates of export of this isoform as observed in Fig. 2.

The ER is typically reticular in structure as shown by with staining of untransfected cells with calreticulin (Fig. 4b). The “donut-shaped” structures resemble the large intracellular structures or intracellular myelin-like figures observed with a mutant form of another myelin protein, peripheral myelin protein-22 (PMP-22) (Dickson *et al.* 2002). The mutant form of PMP-22 is retained in the ER, causing sequestration of another ER chaperone calnexin, and is associated with Charcot-Marie-Tooth-related neuropathies. In MOG 20.5 expressing cells, we find that the distribution of the ER chaperone calreticulin was altered. These structures may be caused by misfolding of MOG 20.5 and the sequestration of calreticulin. Some of the MOG 21.0 that we initially found in a reticular pattern in the ER (Fig. 3) could also be observed in these “donut-shaped” structures in the ER (Fig. 4b).

### Similar subcellular localisations are observed in the oligodendrocyte cell line MO3.13

**Fig. 5:**
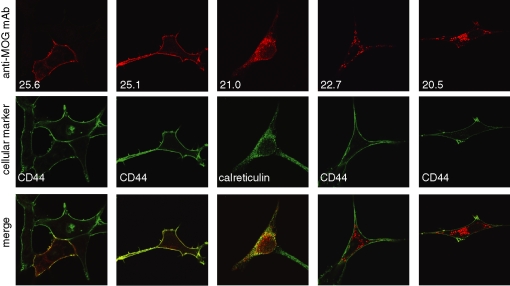
Myelin oligodendrocyte glycoprotein (MOG) isoforms exhibit distinct cellular distribution in MO3.13 oligodendrocyte cells. Confocal micrographs of MO3.13 oligodendrocyte cells transiently expressing MOG isoforms. Cells were treated in acetone before staining with 8.18C5 and Alexa secondary antibodies.

To determine if the striking differences in trafficking, that we observed in HeLa cells, were also found in oligodendrocytes, we transfected the various MOG constructs into MO3.13 cells, an immature oligodendrocyte cell line that does not endogenously express MOG (McLaurin *et al.* 1995; Buntinx *et al.* 2003). These data revealed a very similar pattern of distribution to that observed in HeLa cells. MOG 25.6 and 25.1 were expressed on the cell surface in MO3.13 cells (Fig. 5). MOG 21.0 showed a reticular pattern of expression, which was confirmed to be the ER (Fig. 5). MOG 22.7 was expressed mainly in distinct dots, while MOG 20.5 was expressed at the cell surface with large “donut-shaped” structures (Fig. 5).

### RNA Expression of myelin oligodendrocyte glycoprotein isoforms

**Fig. 6:**
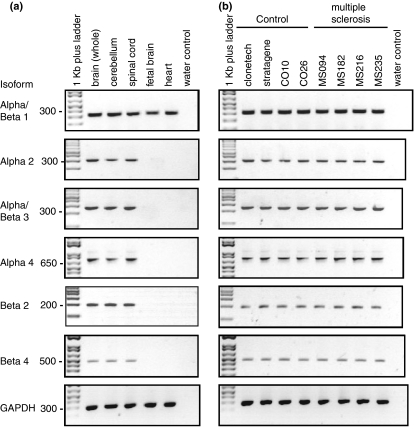
RNA expression of myelin oligodendrocyte glycoprotein (MOG) isoforms. Expression of MOG alternative splice variants, as analysed by svPCR in a) the Human Multiple Tissue cDNA Panels and b) brain (cerebrum) tissue of normal controls and individuals with multiple sclerosis. None of the MOG variants were detected in the following tissues (not shown); adrenal gland, liver, bone marrow, foetal liver, kidney, lung, placenta, prostate, salivary gland, skeletal muscle, spleen, testis, thymus, thyroid, trachea, uterus, colon, small intestine and stomach. Results from corresponding *GAPDH* rt-PCR are shown below the MOG results.

Complex transcription patterns exist for other myelin protein genes, such as myelin basic protein and proteolipid protein genes, with isoforms that show tissue specificity between the nervous and immune systems (Givogri *et al.* 2000). These myelin protein genes clearly have additional functions in a number of different cell types that are unrelated to myelination. This led us to perform a wide-ranging screen for expression of MOG alternative splicing using svPCR in various human tissues (adrenal gland, liver, bone marrow, fetal liver, kidney, lung, placenta, prostate, salivary gland, skeletal muscle, spleen, testis, thymus, thyroid, trachea, uterus, colon, small intestine and stomach), blood fractions and in brain tissue (cerebrum – normal appearing white matter) from individuals with multiple sclerosis and from controls. We observed the expression of full-length variants (MOG 25.1/25.6) in CNS tissues including fetal brain (Fig. 6a). However, MOG expression was not detected in any of the peripheral tissues examined except for the heart, in which we detected the full-length transcripts (MOG 25.1/25.6). This expression most likely originates from myelinated nerves (e.g. vagus fibres) within the heart, although non-myelinating cell types cannot be discounted.

In contrast, expression of the alternative transcripts (MOG 20.5, MOG 20.2, MOG 22.2, MOG 22.7, MOG 16.3 alpha 4 and MOG16.3 beta 4) was limited to adult CNS tissues with no detectable expression of these transcripts in either fetal brain, heart or other human tissue screened. All the MOG variants including full-length (MOG 25.1/25.6, MOG 20.5, MOG 20.2, MOG 22.2/22.7, MOG 16.3 alpha 4 and MOG 16.3 beta 4) were detectable in brain tissue (normal appearing white matter) samples from individuals with multiple sclerosis (Fig. 6b). None of the MOG variants were detected in the following blood fractions: mononuclear cells (B- & T-cells and monocytes), resting CD8+ cells (T-suppressor/cytotoxic), resting CD4+ cells (T-helper/inducer), resting CD14+ cells (monocytes), resting CD19+ cells, activated mononuclear cells, activated CD4+ cells, activated CD8+ cells, activated CD19+ cells.

## Discussion

Several alternatively spliced mRNA transcripts arise from the human MOG gene, and most, if not all, of these are capable of being translated into protein. In this study, we have shown that variation in the cytoplasmic tail of MOG significantly affects the rate of export and the site of expression of MOG proteins (see Table 2 for summary of cellular distribution). Efficient anterograde trafficking of membrane-bound forms of MOG to the cell surface appears to be dependent on having a VLGP×V motif, encoded by exon 7 and 8 and found in MOG 25.6, 25.1 and 22.7. Internalisation of MOG into the endocytic system appears to be promoted by the 10B tail, encoding amino acids LFHLEALSG, in the absence of exon 8, as observed for 21.0 and 22.7. Such intrinsic alternative splicing machinery may operate to regulate the surface expression of MOG. In addition we have shown that MOG 16.3 can be secreted when expressed in HeLa cells. As this may have important implications for the pathogenesis of MS, the confirmation of secreted isoforms of MOG in humans, such as by immunoprecipitation of MOG from cerebrospinal fluid, is required.

**Table 2:** Summary of MOG isoform localisation

Isoform	Cellular localisation	Glycosylation
25.6	Surface	Endo H resistant
25.1	Surface	Endo H resistant
21.0	Mainly ER. Internalised into endosomes	Endo H sensitive
22.7	Surface and endosomes	Endo H resistant
20.5	Mainly ER. Low surface expression	Endo H sensitive
16.3	Secreted	

Using svPCR we detected full-length transcripts of MOG (MOG 25.6 and 25.1) in fetal brain whereas alternative variants were not found. These results are consistent with previous observations and with the implication that the role of full-length isoforms might be specific to myelin maturation (Ballenthin and Gardinier 1996; Delarasse *et al.* 2006). Many of the proposed functions of MOG have been based on its surface localisation on oligodendrocytes. However, secreted MOG released into the cerebrospinal fluid could allow alternative interactions at distant sites. Expression of MOG in the ER and endocytic system also expands the potential functions and interactions of MOG. In addition to a role in cellular transport, cytoplasmic tail residues are important in signalling. Ligation of surface expressed MOG induces signalling cascades resulting in the induction of survival signals, changes in cytosketal integrity or the activation of stress-related pathways (Marta *et al.* 2005b). However, there is potential for secreted MOG to mask such MOG-induced responses. In addition, ligation of MOG with alternative cytoplasmic tails could have profound effects on these events and may trigger alternative responses. With the recent identification of another 11 MOG transcripts, alternative sites of expression, function and signalling events of MOG products unquestionably arise (Delarasse *et al.* 2006).

MOG is an important antigen in MS pathogenesis, activating both T and B lymphocyte responses. Although low levels of MOG have been detected in the mouse and rat thymus (Delarasse *et al.* 2003; Pagany *et al.* 2003), in agreement with Bruno *et al.,* we did not detect the expression in the human thymus (Bruno *et al.* 2002). Lack of expression of MOG in thymus could affect central tolerance, as demonstrated for other antigens.

Differential expression of MOG variants may influence protection from or susceptibility to disease. B cell responses to MOG are considered key to the demyelination of oligodendrocytes (Iglesias *et al.* 2001). Secreted forms of MOG could interact with these demyelinating antibodies and complement, potentially inhibiting their pathogenic effect on oligodendrocytes. The death of oligodendrocytes, proposed to be the first trigger in relapsing and remitting MS (Barnett and Prineas 2004), could expose novel MOG epitopes from the cytoplasmic tail or may reveal alternatively glycosylated forms of MOG, which could be recognised by pathogenic antibodies to the protein (Marta *et al.* 2005a). The observation of slow export and ER accumulation of specific isoforms (such as MOG 20.5) also raises the possibility that the misfolding of MOG and consequential ER stress could be important in certain CNS associated diseases.

Our analysis of brain tissue samples from individuals with MS did not demonstrate any disease-specific differences in splice patterns for MOG in normal appearing white matter. Analysis using adequate sample size and carefully controlled real-time svPCR or oligonucleotide hybridisation arrays is now necessary to determine whether potential quantitative shifts in expression of alternative transcripts exist between disease and non-disease states, e.g. in MS plaques, or in different compartments of the CNS.

## Notes

Whilst this paper was in preparation Allamargot and Gardinier reported that alternative MOG transcripts are translated into protein in human CNS myelin (Allamargot C. and Gardinier M.V. (2007) Several MOG isoforms are expressed in CNS tissue due to alternative splicing of transcripts from the human MOG gene. *J. Neurochem*. **101**, 298–312). Our results are consistent with their findings.

## Abbreviations used

CNS: central nervous system
ER: endoplasmic reticulum
GFP: green fluorescent protein
MOG: myelin oligodendrocyte glycoprotein
MS: multiple sclerosis
PBS: phosphate-buffered saline
PMP: peripheral myelin protein
SDS: sodium dodecyl sulphate
svPCR: splice variant-specific PCR
TM: transmembrane

## References

[b1] Ballenthin PA, Gardinier MV (1996). Myelin/oligodendrocyte glycoprotein is alternatively spliced in humans but not mice. J. Neurosci. Res..

[b2] Barnett MH, Prineas JW (2004). Relapsing and remitting multiple sclerosis: pathology of the newly forming lesion. Ann. Neurol..

[b3] Brunner C, Lassmann H, Waehneldt TV, Matthieu JM, Linington C (1989). Differential ultrastructural localization of myelin basic protein, myelin/oligodendroglial glycoprotein, and 2′,3′-cyclic nucleotide 3′-phosphodiesterase in the CNS of adult rats. J. Neurochem..

[b4] Bruno R, Sabater L, Sospedra M, Ferrer-Francesch X, Escudero D, Martinez-Caceres E, Pujol-Borrell R (2002). Multiple sclerosis candidate autoantigens except myelin oligodendrocyte glycoprotein are transcribed in human thymus. Eur. J. Immunol..

[b5] Buntinx M, Vanderlocht J, Hellings N, Vandenabeele F, Lambrichts I, Raus J, Ameloot M, Stinissen P, Steels P (2003). Characterization of three human oligodendroglial cell lines as a model to study oligodendrocyte injury: morphology and oligodendrocyte-specific gene expression. J. Neurocytol..

[b6] Daubas P, Pham-Dinh D, Dautigny A (1994). Structure and polymorphism of the mouse myelin/oligodendrocyte glycoprotein gene. Genomics.

[b7] Delarasse C, Daubas P, Mars LT (2003). Myelin/oligodendrocyte glycoprotein-deficient (MOG-deficient) mice reveal lack of immune tolerance to MOG in wild-type mice. J. Clin. Invest..

[b8] Delarasse C, Della Gaspera B, Lu CW, Lachapelle F, Gelot A, Rodriguez D, Dautigny A, Genain C, Pham-Dinh D (2006). Complex alternative splicing of the myelin oligodendrocyte glycoprotein gene is unique to human and non-human primates. J. Neurochem..

[b9] Della Gaspera B, Pham-Dinh D, Roussel G, Nussbaum JL, Dautigny A (1998). Membrane topology of the myelin/oligodendrocyte glycoprotein. Eur. J. Biochem..

[b10] Dickson KM, Bergeron JJ, Shames I, Colby J, Nguyen DT, Chevet E, Thomas DY, Snipes GJ (2002). Association of calnexin with mutant peripheral myelin protein-22 ex vivo: a basis for “gain-of-function” ER diseases. Proc. Natl Acad. Sci. USA.

[b11] Gardinier MV, Amiguet P, Linington C, Matthieu JM (1992). Myelin/oligodendrocyte glycoprotein is a unique member of the immunoglobulin superfamily. J. Neurosci. Res..

[b12] Givogri MI, Bongarzone ER, Campagnoni AT (2000). New insights on the biology of myelin basic protein gene: the neural-immune connection. J. Neurosci. Res..

[b13] Hilton AA, Slavin AJ, Hilton DJ, Bernard CC (1995). Characterization of cDNA and genomic clones encoding human myelin oligodendrocyte glycoprotein. J. Neurochem..

[b14] Iglesias A, Bauer J, Litzenburger T, Schubart A, Linington C (2001). T- and B-cell responses to myelin oligodendrocyte glycoprotein in experimental autoimmune encephalomyelitis and multiple sclerosis. Glia.

[b15] Johns TG, Bernard CC (1999). The structure and function of myelin oligodendrocyte glycoprotein. J. Neurochem..

[b16] Kroepfl JF, Viise LR, Charron AJ, Linington C, Gardinier MV (1996). Investigation of myelin/oligodendrocyte glycoprotein membrane topology. J. Neurochem..

[b17] Linington C, Bradl M, Lassmann H, Brunner C, Vass K (1988). Augmentation of demyelination in rat acute allergic encephalomyelitis by circulating mouse monoclonal antibodies directed against a myelin/oligodendrocyte glycoprotein. Am. J. Pathol..

[b18] Litzenburger T, Fassler R, Bauer J, Lassmann H, Linington C, Wekerle H, Iglesias A (1998). B lymphocytes producing demyelinating autoantibodies: development and function in gene-targeted transgenic mice. J. Exp. Med..

[b19] Marta CB, Oliver AR, Sweet RA, Pfeiffer SE, Ruddle NH (2005a). Pathogenic myelin oligodendrocyte glycoprotein antibodies recognize glycosylated epitopes and perturb oligodendrocyte physiology. Proc. Natl Acad. Sci. USA.

[b20] Marta CB, Montano MB, Taylor CM, Taylor AL, Bansal R, Pfeiffer SE (2005b). Signaling cascades activated upon antibody cross-linking of myelin oligodendrocyte glycoprotein: potential implications for multiple sclerosis. J. Biol. Chem..

[b21] McLaurin J, Trudel GC, Shaw IT, Antel JP, Cashman NR (1995). A human glial hybrid cell line differentially expressing genes subserving oligodendrocyte and astrocyte phenotype. J. Neurobiol..

[b22] Pagany M, Jagodic M, Bourquin C, Olsson T, Linington C (2003). Genetic variation in myelin oligodendrocyte glycoprotein expression and susceptibility to experimental autoimmune encephalomyelitis. J. Neuroimmunol..

[b23] Pham-Dinh D, Mattei MG, Nussbaum JL, Roussel G, Pontarotti P, Roeckel N, Mather IH, Artzt K, Lindahl KF, Dautigny A (1993). Myelin/oligodendrocyte glycoprotein is a member of a subset of the immunoglobulin superfamily encoded within the major histocompatibility complex. Proc. Natl Acad. Sci. USA.

[b24] Pham-Dinh D, Della Gaspera B, Kerlero de Rosbo N, Dautigny A (1995). Structure of the human myelin/oligodendrocyte glycoprotein gene and multiple alternative spliced isoforms. Genomics.

[b25] Schluesener HJ, Sobel RA, Linington C, Weiner HL (1987). A monoclonal antibody against a myelin oligodendrocyte glycoprotein induces relapses and demyelination in central nervous system autoimmune disease. J. Immunol..

[b26] Scolding NJ, Frith S, Linington C, Morgan BP, Campbell AK, Compston DA (1989). Myelin-oligodendrocyte glycoprotein (MOG) is a surface marker of oligodendrocyte maturation. J. Neuroimmunol..

[b27] Sospedra M, Martin R (2005). Immunology of multiple sclerosis *. Annu. Rev. Immunol..

[b28] Storch MK, Stefferl A, Brehm U, Weissert R, Wallstrom E, Kerschensteiner M, Olsson T, Linington C, Lassmann H (1998). Autoimmunity to myelin oligodendrocyte glycoprotein in rats mimics the spectrum of multiple sclerosis pathology. Brain Pathol..

[b29] Trowsdale J (2001). Genetic and functional relationships between MHC and NK receptor genes. Immunity.

